# New Perspectives on Chinese Herbal Medicine (Zhong-Yao) Research and Development

**DOI:** 10.1093/ecam/neq056

**Published:** 2011-03-10

**Authors:** Si-Yuan Pan, Si-Bao Chen, Hong-Guang Dong, Zhi-Ling Yu, Ji-Cui Dong, Zhi-Xian Long, Wang-Fun Fong, Yi-Fan Han, Kam-Ming Ko

**Affiliations:** ^1^Department of Pharmacology, Beijing University of Chinese Medicine, Beijing 100102, China; ^2^Department of Applied Biology & Chemical Technology, Hong Kong Polytechnic University, China; ^3^Hôpital de la Tour, 1217 Geneva, Switzerland; ^4^Center for Cancer and Inflammation Research, School of Chinese Medicine, Hong Kong Baptist University, China; ^5^Department of Essential Medicines and Pharmaceutical Policies, World Health Organization, China; ^6^Department of Biochemistry, Hong Kong University of Science & Technology, China

## Abstract

Synthetic chemical drugs, while being efficacious in the clinical management of many diseases, are often associated with undesirable side effects in patients. It is now clear that the need of therapeutic intervention in many clinical conditions cannot be satisfactorily met by synthetic chemical drugs. Since the research and development of new chemical drugs remain time-consuming, capital-intensive and risky, much effort has been put in the search for alternative routes for drug discovery in China. This narrative review illustrates various approaches to the research and drug discovery in Chinese herbal medicine. Although this article focuses on Chinese traditional drugs, it is also conducive to the development of other traditional remedies and innovative drug discovery.

## 1. Introduction

The process of chemical drug discovery is long and arduous that it begins from the search of a potential candidate to the development of a marketable drug. It can span the course of more than a decade, and can cost an average of 800 million USD in the USA [1–3]. Under special circumstances such as the search for effective drugs to treat AIDS, the Food and Drug Administration (FDA) in USA and other countries have encouraged an abbreviated process for drug testing and approval (called fast tracking) [4, 5]. However, the rapid withdrawal of natalizumab used for the treatment of relapsing multiple sclerosis has raised concerns over the issue of commercial licensing [6]. Recently, the number of drugs launched in the market did not increase. From 2006 to 2008, only 30–41 new drugs, including synthetic chemical drugs and biopharmaceuticals as well as new diagnostic agents, reached their first markets [7–9].

Chinese herbal medicine (CHM, Zhong-Yao), a pharmaceutical part of traditional Chinese medicine (TCM, Zhong-Yi), has a long history of use, with extensive literature and clinical applications covering thousands of years [10, 11]. It has been widely accepted that CHM has evolved over the millennia, with a battery of herbal materials to preserve health, to treat and prevent illnesses [12, 13]. Over the past decades, CHM has been an area of intensive research aiming at developing new drugs for the ever-evolving diseases afflicting mankind [14]. Because the development of new chemical drugs remains time consuming, capital-intensive and risky (i.e. a low rate of success), much more effort has been put in CHM for drug discovery. At the present time, CHM has been a novel basis of drug development in China. Up to 2007, China has collected 3563 extracts, 64 715 compositions, and 5000 single compounds from 3000 Chinese herbs, together with about 130 kinds of chemical drugs obtained from either CHM ingredients or their derivatives [15]. In this article we systematically summarized the contemporary approaches and strategies of research and development (R&D) on CHM. We also enthusiastically put forward our point of view on this matter.

## 2. Current Status on the CHM R&D

CHM is used by a large number of patients in China and elsewhere in the world. Currently, CHM is available in both traditional and modern forms of application. Based on some successful experiences, approaches in CHM R&D can be summarized in the following.

### 2.1. Reform of CHM Formulation

Dosage forms of Chinese herbal formula commonly used in clinic include decoction (Tang-Ji in Chinese), powder (San-Ji in Chinese), bolus (Wan-Ji in Chinese), and paste (Jin-Gao in Chinese) and so forth. It was said that Yi Yin, the minister of the Shang Dynasty (1600–1046 BC), invented the decoction of CHM [16]. The manufacture of CHM products has been improved by contemporary pharmaceutical processes, wherein active ingredients from herbs are extracted and concentrated, which are then formulated into granules or easy-to-swallow tablets and oral liquid, and even liquid dosage form for intravenous injection [17–19]. Up to now, China has produced the modern CHM formulations 40 species, >8000 varieties of CHM have been exported to more than 130 countries and regions [20]. In addition, delivery systems for CHM have recently undergone great improvement along with advances in modern pharmaceutical technology. As such, CHM, like Western medicine, can be delivered to specific target and/or exists in controlled release formulation [21–23].

A good example is Xiao-Chai-Hu-Tang, which is comprised of Radix Bupleuri, Radix Scutellariae, Rhizome Pinelliae, Radix Zingiber Officinale, Radix Ginseng, Fructus Jujubae and Radix Glycyrrhizae. These herbal drugs are manufactured and formulated into granules by advance pharmaceutical processes in Japan, which can then satisfy the consumers' need and expectation for quality assurance [24–26]. The manufacture of granule form of CHM is generally described as follows. Raw herbal materials are cut into thin pieces followed by the extraction with hot water. After the filtration process, the water extract is concentrated in order to enhance the stability of chemical ingredients. Finally, the concentrated extract is granulated and packed [27]. However, the price of the granulated form of CHM is twice that of the raw herb, and therefore the former is unwelcome in some poverty-stricken areas in China [28]. There are 51 kinds of CHM granules recorded in *The Chinese Pharmacopoeia 2005*.

Granule forms of CHM (single herb or multi-component formula) offer many advantages over decoction in terms of convenience in administration and efficiency in extraction. The household process of decoction can extract 45% of active ingredients from herbs through simmering [29]. Studies indicated that the extraction efficiency by various methods is in a descending order of pressurized liquid extraction > sonication extraction > supercritical fluid extraction > hydrodistillation and decoction [30]. As such, the traditional way of making decoction of Chinese herbal formula should be modified by using modern pharmaceutical processing technology such as suspension freeze-concentration, progressive freeze-concentration, single- or multi-effect evaporation with an external natural circulating flow, on-line foul preventing evaporation with vapor-liquid-solid flow, reverse osmosis concentration, membrane distillation, osmotic distillation and macro-porous resin adsorption, and so forth [31, 32]. Recently, nanotechnology has been utilized to formulate herbal preparations into nano-sized granules and thereby increases their efficacy, presumably through enhancing the bioavailability [33–35]. The utilization rate of “nanomized CHM” may be achieved up to 95%, and this makes economical sense for expensive CHM. “CHM granules” or “nanomized CHM” are dissolved in hot water and be consumed. The modernized dosage forms represent a convenient and effective way of taking CHM as compared with herbal decoction.

### 2.2. Reform of CHM Formulae

In the practice of TCM, multiple-herb formula is prescribed for patients. According to the theory of TCM, component herbs in a formula can somehow achieve synergistic therapeutic actions and/or minimize the adverse reactions arising from some ingredients [36]. TCM is an ancient medical practice which uses different treatment modalities, such as acupuncture, Qi-Gong, food therapy, touch and massage therapy and CHM therapy, for the prevention and treatment of diseases. Ancient Chinese philosophical concepts on Yin-Yang and Wu-Xing (five elements) form the basis of TCM theories. CHM includes 11 146 kinds of medicinal plants, 1581 kinds of animal drugs, 80 kinds of mineral drugs. There are also more than 50 kinds of processed remedies and 5000 clinically proven folk medicines [14].

In China, there are tens of thousands of herbal prescriptions from CHM, including 84 464 traditional formulae and 10 704 commonly used formulae [37, 38]. There are also herbal formulae from other ethnical medicines such as Dai medicine, Tibetan medicine, Yi medicine, Miao medicine and Naxi medicine, and so forth. They are ready for exploitation in drug discovery. Although TCM, the mainstream of healthcare in China for several thousand years, has accumulated lots of clinical experience, it was developed in ancient time after all. The reform of traditional herbal formulae is necessary in order to make them more applicable to contemporary clinical conditions. For example, Su-Xiao-Jiu-Xin-Wan, which is widely used in China for angina pectoris, is comprised of eight components, including toad venoms, bezoar, moschus, orpiment, borneol, pearl, ginseng and antelope horn [39]. The original formula of Su-Xiao-Jiu-Xin-Wan is Liu-Shen-Wan, consisting of six herbs, namely, bezoar, moschus, toad venoms, orpiment, borneol and pearl.

Nevertheless, the synergistic interactions between component herbs are not always obvious. Su-Bing-Di-Wan, comprising two herbs (styrax and borneol), is derived from a parent formula Su-He-Xiang-Wan containing 15 herbal components. However, both formulae displayed comparable clinical efficacy on coronary heart disease [40]. The use of a simplified formula can certainly lower the drug cost, reduce the rate of drug adverse reactions and conserve Chinese herbal resources. In an herbal formula, the overall efficacy of two or more than two component herbs may not always be more than additive, that is, >2. Sometimes, adverse interactions may occur.

### 2.3. CHM-Derived New Chemical Composition

The chemical composition of naturally grown herb may vary according to climatic conditions, harvest time, storage condition, and so on. As such, the same type of herb can vary in its composition and concentrations of chemical constituents from batch to batch. These variabilities can result in significant differences in pharmacological activity [41]. Therefore, the identification and extraction of active ingredient(s)/chemical group(s) from an herbal remedy represents a new approach in the development of CHM. The herb-derived chemical drugs, which have well defined pharmacodynamic and pharmacokinetic profiles, are manufactured in pharmaceutical grade with standardized chemical composition. One good example is Ginkgo (Yín-Xìng in Chinese) leave extract which contains 24% ginkgo-flavonol glycosides and 6% terpene lactones [42]. At the present time, there are 15 major categories of active ingredients in CHM, including flavones, alkaloids, glucides, glycosides, volatile oils, resins, phytochromes, organic acids, amino acids, tannins, proteins, enzymes, trace elements, polysaccharides and mineral salts. Each phytochemical group contains thousands of single compounds. For example, flavones contain more than 9000 derivatives/analogs of known structures, which produce anti-cancer, anti-oxidant, anti-inflammatory, anti-bacterial, anti-virus, anti-ischemic and anti-hypertensive actions [43, 44]. Inorganic salts present in CHM belong to 25 elements, including Be, Cr, Cu, Zn, Ge, Sr, Mo, Cd, Tl and Pb, and so forth [45].

In China, the pharmaceutical industry mainly focuses on the exploration of active extracts rather than single compound in the product development from CHM. A pure compound isolated from an herb is regarded as chemical drug (also known as Western medicine). While water or ethanol extracts of herbs are too crude in terms of chemical composition, preparations containing concentrated phytochemical compounds are preferred to be used as herbal drugs. For instance, Di'ao-Xin-Xue-Kang (Chinese), a Chinese herbal formula used for the prevention and treatment of coronary heart disease including angina, is consisted of steroidal saponins from *Dioscorea panthaica* Prain et Burkill and *Dioscorea nipponica* Makino [46–48]. Yu-Feng-Ning-Xin-Pian (Chinese), a formula used for the treatment of hypertension, senile cerebrovascular disease and angina pectoris, is consisted of total flavonoids (including puerarin) derived from Radix Puerariae [49, 50].

### 2.4. CHM-Derived New Chemical Compound

Despite the long history of use, many clinical proven Chinese herbal formulae are lack of a well defined mechanism of action. It may therefore be necessary to identify the active ingredient(s) from an herbal extract for mechanistic investigations. Up till now, many active ingredients have been isolated from commonly used CHM such as anti-HIV candidate leucovorin and agaritine derivatives as well as anti-Alzheimer's disease and anti-inflammatory candidate GTS-21 [51, 52]. These compounds may serve as potentially useful drug candidates for development at a lower cost. In this regard, active herbal ingredients, such as alkaloids, flavonoids, quercetin, terpene and lignoids, have been shown to produce anti-HIV action [53]. In addition, huperzine A, a novel alkaloid isolated from *Huperzia serrata*, is clinically used for the treatment of Alzheimer's disease and vascular dementia [54]. Berberine, an active ingredient from *Coptis chinensis* Franch, is widely used for the treatment of infectious diseases in China [55]. Notably, arsenious acid derived from arsenic trioxide (Pi-Shuang in Chinese) has become a first-line drug for the treatment of acute promyelocytic leukemia [56, 57].

With the growing popularity of CHM for the prevention and treatment of diseases, the ever-increasing demand for Chinese medicinal plants has posed a threat to their extinction. The advent in medicinal plant cell culture technology, which offers an effective alternative for supplying desirable compounds in lieu of extraction from natural sources, comes to the rescue. Active ingredients of ginseng, such as saponins and polysaccharides, were obtained from cultured cells of *Panax ginseng* to meet the need for mass production of these bioactive ingredients [58, 59]. Plant cell and tissue culture methods was introduced in the early 1960s, and commercial applications have been underway since the late 1980s [60]. Paclitaxel (taxol) is an effective anti-cancer agent found in *Taxus mairei*. The plant is facing the threat of extinction because of the great demand for wood and taxol production, as well as the difficulty in its propagation. Fortunately, an effective cell culture technology for taxol production has been established [61, 62]. In addition, the chiral property or physical state (particularly for microelements) of the active ingredient of an herb may be an important determinant for its pharmacological and toxicological profiles [63]. For instance, the nootropic action of (−)clausenamide is 10- and 20-fold higher than those of (+)clausenamide [64, 65].

### 2.5. Optimization of CHM-Derived Lead Compound

Active ingredient(s) isolated from CHM can be produced by semi- or total synthesis, with or without structural modification for activity optimization. Given that designing a new chemical entity from scratch is a daunting task, it is possible to develop derivatives based on known molecular structures from various herbal ingredients. In this respect, artemisinin, a powerful anti-malarial drug with a rapid onset of action and low toxicity, was extracted from Qing-Hao (Chinese) in 1971 [66, 67]. At the present time, it is a key drug in combination therapies recommended by the World Health Organization (WHO) for the treatment of uncomplicated multi-drug-resistant strains of *Falciparum* malaria. Dihydroartemisinin is a compound derived from artemisinin with higher clinical efficacy and safety than that of the parent drug [68, 69]. In addition, other Qing-Hao-Su (Chinese) derivatives have been shown to possess anti-parasitic and anti-cancer properties [70]. In China, Qing-Hao for treating malaria has begun in the fourth century [71]. Artemisin derived from Qing-Hao shows once again that natural products serve as an invaluable source of lead compounds for sophisticated small molecule drugs [72].

In an effort to develop novel anti-viral hepatitis agent, a number of analogues of schisandrin C (dimethyl-4,4′-dimethoxy-5,6,5′,6-dimethylene dioxybiphenyl-2,2′-dicarboxylate), an active ingredient isolated from Wu-Wei-Zi (Chinese) were synthesized, including bifendate (dimet hyl-4,4′-dimethoxy-5,6,5′6′-dimethylene-dioxybipheny1-2,2′-dicarboxy late) and bicyclol (4,4′-dimethoxy-5,6,5′,6′-dimethylene-dioxy-2-hydroxymethyl-2′-carbonyl biphenyl) [73, 74]. These compounds, like Wu-Wei-Zi, have been shown to protect the chemically induced hepatotoxicity in experimental animals and to be effective in treating patients with chronic viral hepatitis as well as some other diseases [75–77].

### 2.6. Matching of CHM Ingredients and Molecular Targets of Disease

In the past, most drugs were discovered either by identifying the active ingredient(s) from traditional remedies or by serendipity. A new approach for drug discovery involves the elucidation of molecular mechanism(s) underlying the disease/infection process followed by the identification of drug target. Despite the advances in technology and biomedical sciences, drug discovery is still a time-consuming process, with a low rate of success. Recently, a search of active ingredient(s) from 240 commonly used CHM in relation to relevant drug targets in a database has revealed a wide variety of compounds that have therapeutic potential. About 62% of the species were found to contain chemical compounds with pharmacological profile beneficial for the treatment of at least one disease and 53% of them for two or more diseases. Approximately 7000 unique compounds are listed, though some are found in more than one herb, the total number for all herbs being 8264. For bioactive plant compounds, 2597 compounds active against 78 molecular targets are covered [78]. In comparison with the high-throughput screening against a chosen target for a particular disease, virtual mapping between databases of Chinese herbal ingredients and molecular targets of diseases is likely to offer a new avenue for drug discovery [79–81].

### 2.7. CHM-CHM Interaction in Multi-Component Herbal Formulae

The principle of formulation in TCM is adopted to guide the choice of herbs (herb matching) in multi-component herbal formulae prescribed for the treatment of diseases. It is documented that 91% of the 6986 herbal formulae are multi-component and 3196 herbal materials were used to constitute 11 810 formulae [82]. As such, an optimal therapeutic effect can be achieved by herbal treatment. While the theory underlying the principle of formulation has yet to be clearly established in modern scientific terms, two possible outcomes resulting from herbal interaction in the mixture have been proposed: (i) synergistic interaction among component herbs and (ii) generation of secondary compound(s) from the decoction process. The significance of herb matching has been revealed in a few formulations including Six Ingredient Rehmannia Decoction (Liu-Wei-Di-Huang-Wan) and Pulse Promoting Drink (Sheng-Mai-Yin or its parent formula Shengmai San) [83, 84]. All of these formulae produce a therapeutic action, including chemical entity, which is distinct from that produced by a single component herb [85].

Seeking new chemical entities in multi-component herbal formulae is a promising approach in drug discovery. Up to now, there is no successful example. Since the effects of Chinese herbal formulae are not simply the summation of component herbs, one or more new compound(s) may be produced from herb-herb interaction during the decoction process. These new chemical compounds should be identified.

### 2.8. CHM Metabolites

Pharmacokinetics is a branch of pharmacology specialized in the determination of the fate of drugs following the administration into a living organism. According to pharmacokinetics studies, administered drugs may have their chemical structures altered after metabolism and biological activities of the parent drugs are thus changed [86, 87]. In this connection, the pharmacological/toxic action of drugs may not be due to the drug itself, but to its active metabolite(s) in the body. For instance, the half-life of intravenously administered levosimendan, an inotropic drug, is very short (∼1.5 h), but it can cause a prolonged hemodynamic improvement in patients with heart failure because of its long half-life active metabolite, OR-1896 [88, 89]. In addition, genotoxic metabolites of estradiol can bind onto DNA and cause depurination, thereby developing breast cancer in women [90].

Chinese herbal formulae are consisted of multiple herbs and therefore are liable to produce a large number of metabolites which may act on multiple targets in the body. Availability of such a large number of metabolites is likely to open up opportunities for drug discovery [91]. Pharmacological investigations on herbal formulae and their metabolites may reveal the complexity of inter-organ functional relationship, which is instrumental in defining the therapeutic action of multi-component herbal remedies.

### 2.9. Integrative Drug

In China, CHM can be combined in use with Western drug (i.e. chemical drug). In doing so, Chinese herbal extract or active ingredient can be admixed with Western drug. Despite the difference in the diagnostic and treatment principle between Chinese medicine and Western medicine, over the past 50 years, the integration of these two medical systems in clinical settings has become popular and achieved significant developments in China [92]. This area of integrated medicine is referred to as modern Chinese medicine (MCM). The “marriage” of Chinese medicine and Western medicine has indeed brought a lot of benefits to patients. In most clinical conditions, the combination therapy with CHM and Western drugs provides a greater therapeutic effectiveness than using either herbal or Western drug alone. One clinical condition in which MCM is commonly applied is chronic hepatitis C. The MCM-based treatment for hepatitis C is aimed to (i) control liver inflammation and prevent fibrosis by Western drugs and (ii) restore normal liver functions and overall health condition, particularly the immune function, with herbal remedies [93].

Currently a large number of formulations containing CHM and Western drug are available in China market. There are 201 Chinese herbal preparations (internal medicine 175 kinds, topical medicine 58 kinds) admix with Western drug(s) in *The Chinese Pharmacopoeia 2000*. On the other hand, pure ingredient or its derivative in CHM is mixed with chemical drug. For example, artemether compound tablet (Coartem), which is an anti-malarial agent consisting of artemisinin derivative artemether and synthetic benflumetol [94, 95].

### 2.10. Covalent Modification of CHM Compound with Chemical Drug

Drug developed from MCM may involve the direct structural linkage between Chinese herbal compound and Western drug. For example, huperzine A, a naturally occurring sesquiterpene alkaloid found in *H. serrata*, and tacrine, a prototypical cholinesterase inhibitor, are covalently linked to form a novel dimer, HA′(10)-tacrine [96, 97]. Experimental studies indicated that HA′(10)-tacrine was effective for improving cognitive deficits in several animal models and caused a relatively low liver toxicity [98, 99]. HA′(10)-tacrine may therefore be used for the treatment of Alzheimer's disease and other neurodegenerative disorders. In addition, two Chinese herbal compounds can be linked to form a new drug. Tanshinol borneol ester (1,7,7-trimethylbicyclo [2.2.1] heptan-2-yl-3-(3,4-dihydroxyphenyl)-2-hydroxy-propanoate), a novel compound synthesized by linking tanshinol and borneol ester, is prescribed for the treatment of cardio-cerebrovascular diseases [100].

**Figure 1:**
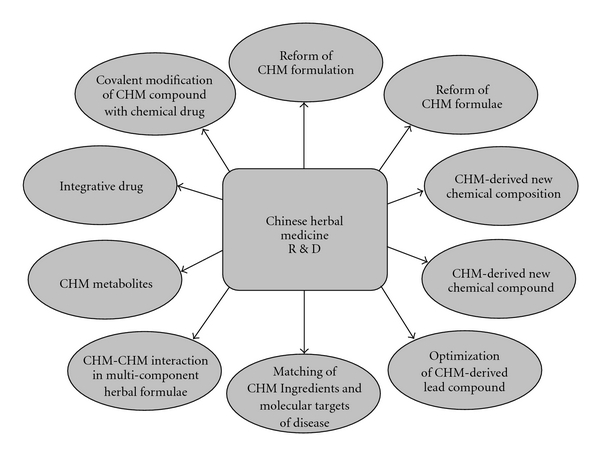
Approaches in R&D of CHM.

Despite the modernization process, the practice of TCM should still be based on the holistic concept of integrated body systems that work in balance to maintain the healthy body condition. With such a mind set in place, drug discovery from CHM should be focused on compounds or extracts that can act on multiple targets in the body. In view of the current technological support and market sentiment, some of the aforementioned approaches should be feasible for drug discovery (Figure 1).

## 3. Discussion

At the present time, many people are interested in CHM including other nature remedy and alternative therapy [101], with an attempt to explore its commercial potential. In doing so, we should note the following points.

### 3.1. An Herbal Remedy Cannot Be Substituted by a Single Compound

Three reasons are likely underlying the increasing popularity of herbs. First, modern medicine has failed to offer a complete cure for many diseases. Secondly, patients are turning to herbs in dealing with symptoms or side effects resulting from chemical drug treatments. Thirdly, people utilize herbs to improve the overall well-being and health or to prevent diseases. With the mind set of modern medicine, once the efficacy of an herb is validated by scientific investigations, scientists will attempt to substitute the herb with its active extract or ingredient. For example, morphine, a naturally occurring compound extracted from Asian opium poppy plant, is not only a very potent analgesic but also a highly addictive drug. In this regard, scientists have attempted to eliminate its addictive property and at the same time, retain its analgesic action through modifying the chemical structure of morphine. As a result, heroin (diamorphine) was made. Methamphetamine hydrochloride (ice, Bing-Du in Chinese) is a product from the structural reformation of ephedrine, an alkaloid found in a CHM, *Ephedra sinensis*. Unfortunately, while drug addicts over the world amount to 25 million, the market for methamphetamines and amphetamines continues to be larger than that for cocaine (14 million users) or heroin (11 million users) [102].

While isolating or concentrating an active ingredient from an herb can markedly enhance its therapeutic potency on a specific aspect, as compared to the use of crude herb, herbal extract, or herbal decoction, it may also lead to undesirable side-effect(s) due to the unbalanced mode of action. The so called “herbal medicine” that uses the active ingredient isolated from an herb or synthesized by chemical processes is not much different from a chemical drug. After all, a single active ingredient from an herb should be regarded as a chemical drug in modern medicine and will be examined according to the Western pharmaceutical compound standard. Therefore, the drug discovery from herbs, particularly CHM, should focus on multi-component herbal formulae (Fang-Ji in Chinese), with emphasis on the herb-herb interaction in producing a therapeutic effect. The FDA recognizes CHM as medicinal products containing active ingredients of exclusively plant origin without well-defined active ingredients [27].

In addition, the active single compound is often unidentifiable and its isolation or extraction is also difficult. Furthermore the combination and matching of more than two herbs in CHM is considered more useful than using a single one. In fact, combination therapy in Western medicine (chemical drug) is also a common practiced in clinical situation such as cocktail therapy in AIDS [103] and combination medication in patients with hypertension [104]. Like chemical drug, there are additive, synergic, antagonistic, inhibitory, destructive and opposite action among herbs or their ingredients. Under the guidance of TCM theory, the combination of herbs would enhance the efficacy and minimize the adverse reaction of CHM. More importantly, pure compound derived from CHM might lose the original properties of the remedy. Unlike Western medicine, the application of CHM prescriptions (formulas) is guided by herbal properties (Yao-Xing in Chinese) and symptoms/signs of patients (Zheng-Hou in Chinese) rather than a well-defined drug target [105, 106].

### 3.2. New Applications of Traditional Herbal Remedy

Since ancient time, plants have been employed as the source of medicine in all cultures. The new use of old herbs, as were the cases for many chemical drugs, is important for introducing new applications for traditional herbs. For instance, Fructus Schisandrae (FS), the fruit of Wu-Wei-Zi, is regarded as a tonic agent in CHM. FS has been used for thousands of years in China. A large number of pharmacological and clinical studies have shown that FS produces a systemic effect in the body [107]. In our laboratory, an ethanol extract of FS [108], its active ingredient schisandrin B [109], as well as its related synthetic derivatives bifendate [110] and bicyclol [111] have been shown to suppress the lipid accumulation in the liver, but without affecting serum lipid levels, in experimental hypercholesterolemic mice. The possible clinical use of FS for the clinical management of fatty liver disease exemplifies the new use of an old herb in CHM. In addition to antimicrobial activity, berberine, an alkaloid present in *C. chinensis* Franch., has been found to possess hypoglycemic and hypocholesterolemic activities as well as other pharmacological properties [112–114].

In addition, veterinary drugs, diagnostic/pharmacological agents for the research in life sciences, and food additives may be developed from herbal remedy. These are often cheaper and easier to develop than new therapeutic agent or compound. Recently, for example, Pan et al. have established an animal model of hypertriglyceridemia using schisandrin B isolated from Fructus Schisandrae and its related synthetic analog bifendate [115, 116].

### 3.3. New Source of Herbal Ingredients

Herbs are derived from plant materials such as leaves, flowers, fruit, seed, stems, wood, bark, roots, rhizomes or other plant parts. Given that the therapeutic effect of an herb depends on the ingredients present in the plant part, if the bioactive substance is also present in other plant parts or even the whole plants, it can be exploited. Ginsenosides, which encompasses more than 40 kinds of monomeric compounds, are major active ingredients of Radix Ginseng (the root part of *P. ginseng*) with a wide spectrum of pharmacological actions [117, 118]. In fact, ginsenosides were also found in the leaves and stems of *P. ginseng* [119, 120]. It is likely that leaves and stems of *P. ginseng* can produce similar pharmacological actions as Radix Ginseng.

For example, tea-drinking is invented by Chinese about 4700 years ago. Saichyo (767–822 AD), a Japanese who studied in China, was the first man bringing tea-drinking to Japan. In 1610, Dutch merchants transported tea from China to Europe [121]. Tea-drinking has been regarded as a health-promoting habit in the world [122–124]. Many plant species other than *Camellia sinensis* have been used for tea leaves. In addition, tea flower is used as food in Japan [125] and oil extracted from tea seeds is used as cooking oil in China [126].

### 3.4. Highlight the Advantages of CHM

In fact, complementary and alternative medicine (CAM) involves drug therapy (pharmaceutical CAM) and non-drug therapy (non-pharmaceutical CAM). Drugs employed by all CAM are herbs including spices and vegetables. Herbal medicine worldwide can be classified into four basic systems, namely, Chinese herbalism, Ayurvedic herbalism [127–129], Western herbalism and Arabic/Islamic herbalism [130, 131]. Western herbal medicine, which was originated from Greece and Rome, had been spread from Europe to North and South America. However CHM is of more multiplicity than other CAM. They include herbs (90%), animal parts, minerals and crude synthetics. When compared with other traditional herbal medicines elsewhere in the world, CHM has been endowed with a more complete set of theories and therapeutic principles from TCM for guiding different treatment modalities in a variety of clinical conditions. Unlike some other traditional medicines, TCM is not attached to any religions or superstitious beliefs. Given the less sophisticated way of making diagnosis and treatment, the practice of TCM, with the use of CHM for treatment, is much less costly than modern medicine for patients. Cost-effective choice in the treatment of disease is important for patients, physicians and policy makers under the times of economic crisis [132]. It is not unusual for TCM practitioners to succeed in treating difficult clinical conditions that modern medicine has failed.

There are many differences between TCM and Western medicine. The prescription of Chinese herbal formula as a treatment modality for diverse medical conditions is based on the differentiation of symptoms and signs, also known as Bian-Zheng in Chinese [12, 133]. According to TCM theory, patients can be categorized into eight groups: Yin or Yang, Biao or Li (superficial or internal), Han or Re (cold or hot), Xu or Shi (deficient or replete). All these disease states may or may not be paralleled by counterparts in Western medicine. Medicinal property of CHM is divided into Si-Qi (four properties) such as cold, cool, warm and hot and Wu-Wei (five tastes) such as pungent, sour, sweet, bitter and salty. The application of CHM in clinical condition is based on the diagnosis derived from Bian-Zheng and medicinal properties of CHM, with the therapeutic aim of re-adjusting the body to a normal condition from a disease state, rather than just treating disease [134].

The practice of TCM is mainly based on an empirical approach which has yet to be accepted in Western medicine. Despite the long history of use, the therapeutic application of CHM or their formulae needs to be validated by experimental and/or clinical evidence obtained from contemporary studies using appropriate methodologies. After all, the efficacy and toxicity of CHM need to be addressed by scientifically acceptable language in the 21st century. Herbal interaction among component herbs in a formula should be analyzed and revealed. In view of these, the CHM R&D is certainly a challenging task.

It is true that most Chinese herbal preparations do not look as pleasant or good in appearance or taste as modern chemical drugs, and their action mechanisms and dosages are not defined in a precise manner. However, with appropriate applications, CHM can work as good as chemical drugs, but with considerably fewer serious adverse effects. CHM is a wealth of naturally occurring bioactive agents. This has, in turn, provided a golden opportunity for drug discovery in CHM. With all “natural forces” at work, the nature will provide a plentiful of exploitable resources for maintaining life activities. Defense against diseases are readily available in the form of naturally occurring chemicals or substances such as CHM.

## 4. Conclusion

Initially driven by chemistry and increasingly guided by pharmacology and clinical sciences, drug research has contributed more than any other scientific factors to the progress of medicine during the past century. Although modern pharmaceutical science has achieved tremendous success in treatment of diseases, including those being regarded as incurable for decades, a considerable number of diseases still remain incurable by chemical drugs. Herbal medicine, particularly CHM, has a traditional history of use, being rooted in the cultural heritage of different ethnic groups. To demonstrate that herbs produce as much beneficial effect as modern medicine by scientific means remains a challenge, particularly when using criteria applied for assessing chemical drugs which are consumed in a purified and concentrated form [135]. Nevertheless, we firmly believe that CHM, with its long history of use and detailed documentation in both theory and practice, will play an important role in new drug discovery in the future. As the theory of TCM goes, we should count on our body defense mechanism for fending off diseases. That means “treat before getting sick”. In this regard, many CHM can be used for up-regulating the functioning of antioxidant and immune systems in the body. If researches on CHM-derived drug are conducted using contemporary methodologies and biomarkers, they will produce great impact on the mainstream biomedical science and likely bring about an era of modern medicine [136, 137].
